# Non-gestational Choriocarcinoma of the Ovary: A Report of a Rare Case From Saudi Arabia

**DOI:** 10.7759/cureus.66487

**Published:** 2024-08-09

**Authors:** Hesham K Sait, Fahad Alghamdi, Yaser Ragab, Sarah Aljadani, Khalid H Sait

**Affiliations:** 1 Obstetrics and Gynecology, Faculty of Medicine, King Abdulaziz University, Jeddah, SAU; 2 Obstetrics and Gynecology, Tom Baker Cancer Centre, University of Calgary, Calgary, CAN; 3 Pathology, Faculty of Medicine, King Abdulaziz University, Jeddah, SAU; 4 Radiology, Dr. Erfan and Bagedo General Hospital, Jeddah, SAU

**Keywords:** perimenopause, non-gestational choriocarcinoma, radiological findings, clinical pathology, ovary

## Abstract

Non-gestational choriocarcinoma of the ovary is extremely rare and presents diagnostic and therapeutic challenges. Early recognition, appropriate surgical intervention, and adjuvant chemotherapy are essential for successful management. This case underscores the importance of considering choriocarcinoma in the differential diagnosis of ovarian tumors, especially in perimenopausal women with vascular mass.

We present the case of a 47-year-old sexually active woman with a history of pelvic pain, diagnosed with non-gestational choriocarcinoma of the ovary. The patient underwent total abdominal hysterectomy and bilateral salpingo-oophorectomy with successful management using the bleomycin, etoposide, and platinum (BEP) regimen. This case highlights the importance of early detection and appropriate management of this rare entity.

## Introduction

Choriocarcinoma is an invasive highly vascular tumor consisting of trophoblastic anaplastic tissue made up of cytotrophoblasts and syncytiotrophoblasts with no chorionic villi. It is a very aggressive tumor, characterized by early vascular invasion and widespread metastasis [1].

Choriocarcinomas can arise from gestational or non-gestational tissues. Most choriocarcinomas are of gestational origin, known as gestational choriocarcinomas (GCC), while non-gestational choriocarcinomas (NGCC) are extremely rare and account for 0.6% of all malignant germ cell tumors [2]. Ovarian NGCC usually arises from the retained primordial germ cells due to faulty migration during embryogenesis. In addition, it may arise due to the aberrant or divergent differentiation of the underlying somatic malignancy [3].

It is imperative to differentiate between GCC and NGCC, as this will help in choosing the right treatment modality (i.e., surgery versus chemotherapy and choice of chemotherapy), thereby affecting the patient's prognosis and outcome. GCC is a highly chemosensitive tumor with a usually favorable prognosis, whereas in NGCC, even with multidrug therapy strategies, a large number (84%) of patients succumb to the disease, indicating resistance to various treatment modalities [4]. Moreover, the staging also differs. It is necessary for deciding the follow-up duration.

Metastatic manifestations such as central nervous, cardiopulmonary, and gastrointestinal can be present [5]. The secretion of human chorionic gonadotropin (HCG) may lead to menstruation abnormalities and puberty disorders in young children and ovarian hyperstimulation in reproductive-age women. The ambiguity of the symptoms can render establishing a timely diagnosis quite challenging and lead to delayed treatment [2,6]. We report a rare case of NGCC of the ovary in a perimenopausal woman.

## Case presentation

**Figure 1:**
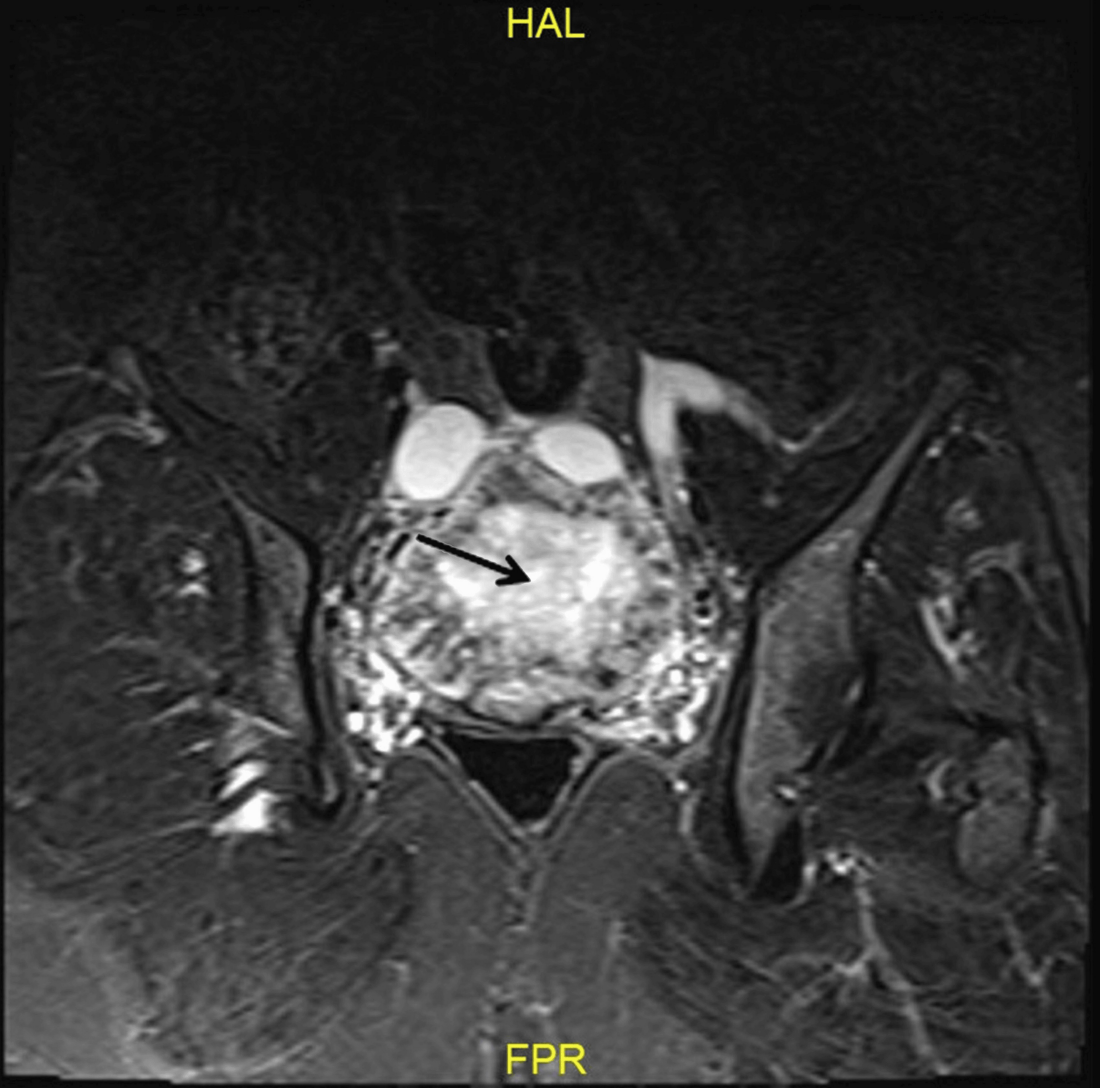
Coronal T2WI showing the mass with engorged bilateral pelvic veins

**Figure 2:**
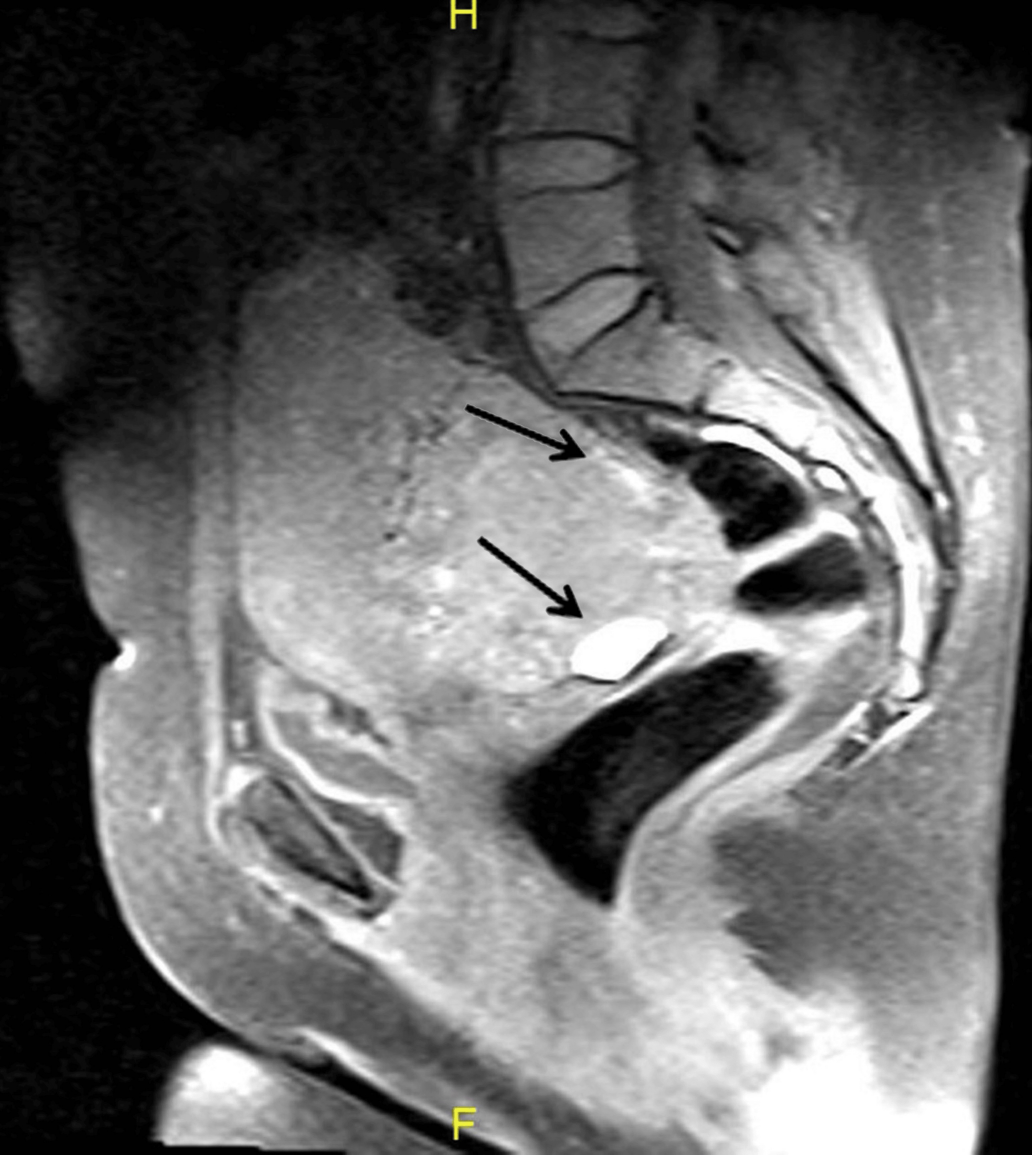
Post-contrast sagittal T1 fat saturation showing heterogeneous enhancement and high signal components of subacute blood

**Figure 3:**
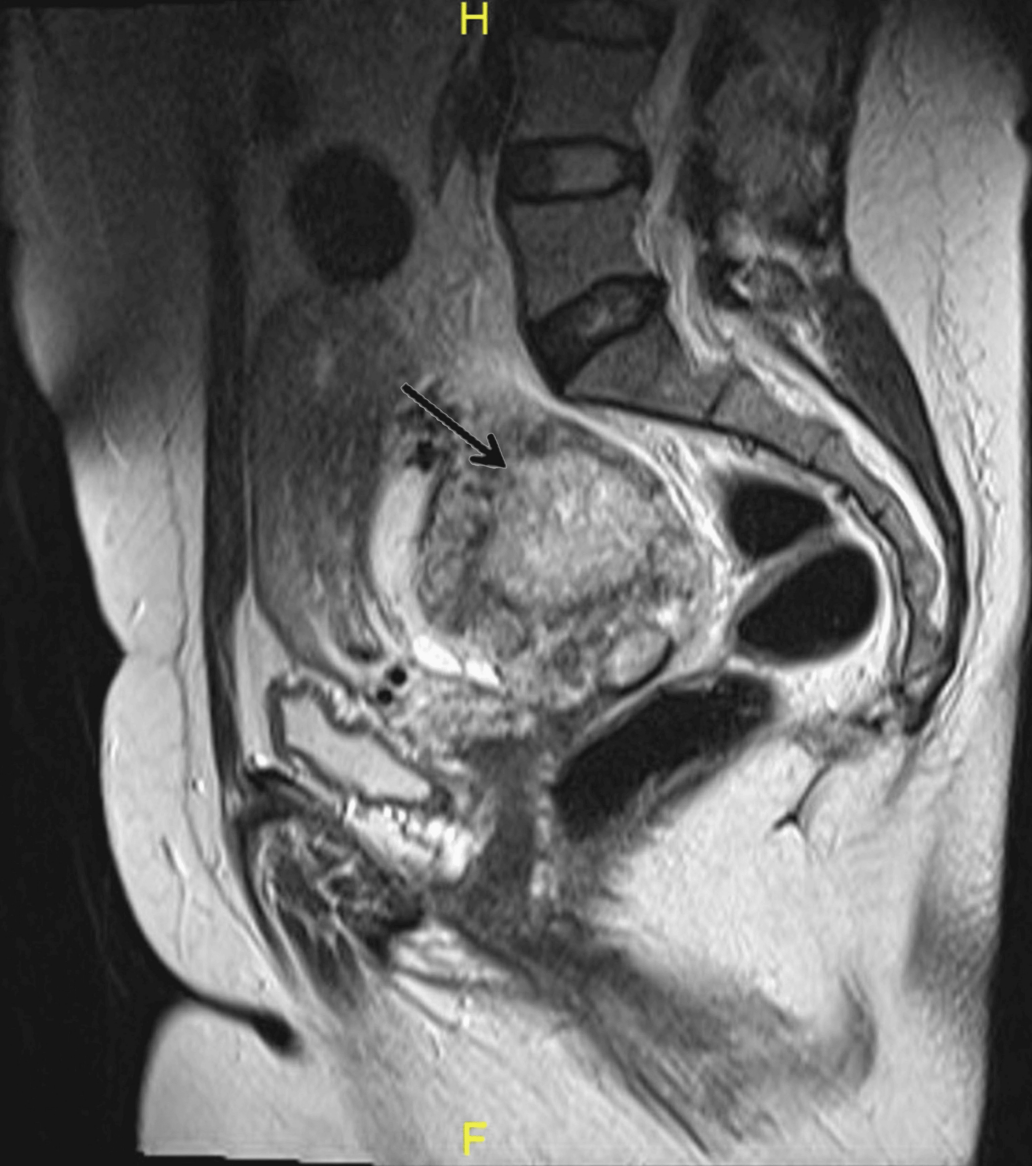
Sagittal T2WI showing heterogeneous hypo- and hypersignal mass lesion

A 47-year-old para 5 sexually active woman presented with a complaint of pelvic pain for the last two months. She had a history of dilation and curettage one year prior with histopathology showing products of conception. Since that time, her menstrual period has been irregular. Ultrasound of the pelvis showed a right large ovarian mass with increased vascularity. Magnetic resonance imaging (MRI) revealed a complex right ovarian mass, measuring 7×10×8 cm (Figure 1, Figure 2, and Figure 3) with no evidence of distal metastasis.

Her tumor markers were normal apart from an elevated HCG level of 305,000 mIU/mL (Table 1).

**Table 1:** Finding of tumor markers in the reporting case HCG: human chorionic gonadotropin; LDH: lactate dehydrogenase

Tumor markers	Result	Normal value
CA125	4.3 U/mL	0-35 U/mL
HCG	305,000 mIU/mL	<15 mIU/mL
Alpha-fetoprotein	3.2 ng/ml	0-10 ng/ml
LDH	38 IU/L	48-115 IU/L

**Figure 4:**
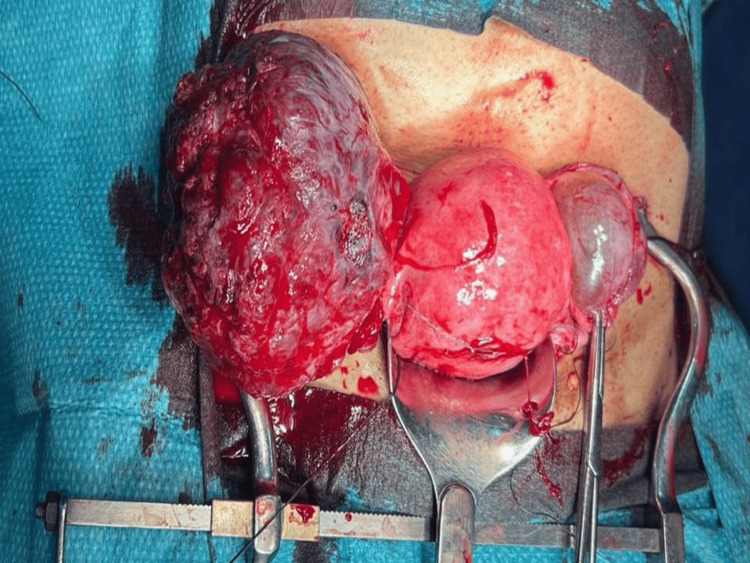
Intraoperative finding of the right large vascular ovarian mass

The patient was referred to a gynecology oncology service, extensive counseling was done, and she underwent laparotomy which revealed a large vascular right ovarian mass (Figure 4). Total abdominal hysterectomy and bilateral salpingo-oophorectomy were performed with no residual disease.

Pathology showed mononucleated and multinucleated trophoblastic cells in varying proportions. The cytotrophoblasts and intermediate trophoblasts exhibit small, round nuclei that are densely stained (hyperchromatic). Their cytoplasm appears clear to eosinophilic (pink-staining) in color, and their cell borders are distinct.

In contrast, the multinucleated cells are larger and have irregular outlines. These correspond to the syncytiotrophoblasts. The tumors show extensive areas of hemorrhage and necrosis. 

**Figure 5:**
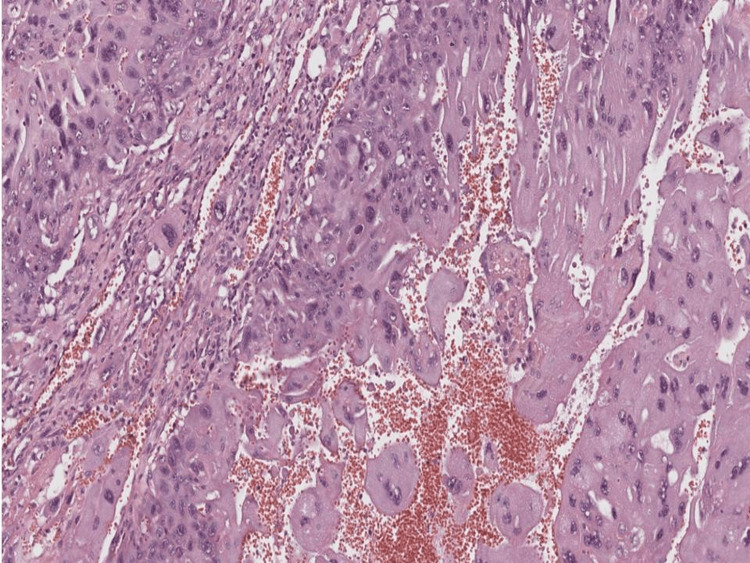
Histological examination unveils the presence of an infiltrative solid tumor characterized by atypical syncytiotrophoblast, cytotrophoblast, and intermediate trophoblast. The absence of chorionic villi is noted. Numerous mitotic figures are discernible within the tissue. Concurrently, the background exhibits regions of necrosis and hemorrhage

The above findings were consistent with the diagnosis of NGCC of the ovary that has ovarian capsule involvement (Figure 5). The patient was treated with four cycles of chemotherapy that included bleomycin, etoposide, and platinum (BEP). With follow-up HCG during treatment, she responded well to the BEP regimen with normalization of HCG levels after treatment. She was followed up for two years with no recurrence.

## Discussion

Differentiating between GCC and NGCC can be challenging; both have distinct entities, with significant differences in their etiology, pathogenesis, clinical presentation, management, and prognosis. A clinical history of pregnancy, amenorrhea, or antecedent gestational trophoblastic disease helps to establish the gestational or non-gestational nature of choriocarcinoma [7].

It has been suggested that the diagnosis of NGCC was based solely on a long interval from the onset of cancer from the index pregnancy [8].

The interval from index pregnancy to presentation can be quite long in GCC as well and hence should not be the sole differentiating criterion. Both GCC and NGCC are associated with elevated serum levels of HCG, thereby causing a diagnostic conundrum. Even though our patient had an abortion one year prior to her diagnosis, NGCC was diagnosed. This sheds light on the importance of understanding the pathological course of such aggressive cancer. NGCC frequently affects teenagers and women of early reproductive age. Liu et al. found that its peak incidence occurs in adolescents aged 12-25 years [9]. However, a few cases of NGCC have been reported in the postmenopausal period [10-12]. Our patient was close to her menopausal age and presented with pelvic pain for two months, which is a vague nonspecific symptom like most ovarian cancers. Vaginal bleeding and abdominal pain have been reported to be the most common symptoms of NGCC [6].

Ovarian choriocarcinoma can present with nonspecific signs and symptoms such as abdominal pain, bloating, adnexal mass, hemoperitoneum, and vaginal bleeding which can mimic an ectopic tubal pregnancy in adults [8].

The initial imaging of choice is pelvic ultrasound which might show a unilateral, very rarely bilateral, solid echogenic and non-homogenous mass with a normal-appearing uterus and endometrial thickness. On color Doppler, intense vascularity with low resistance arterial waveform can be seen [6,13].

Further CT or MRI is important to evaluate the extent of the disease. The mass will appear as well-defined and hypodense with intense post-contrast enhancement [13]. Due to the natural property of trophoblastic cell of invasion, hemorrhagic lesions in different metastatic sites can be detected and lead to non-gynecological bleeding symptoms [6]. Staging for NGGC is still not agreed upon. Multiple studies reported using either the 2013 FIGO staging of ovarian cancer or the 2010 FIGO staging of choriocarcinoma [12-14].

Histomorphologically, both GCC and NGCC are the same. To date, no single or established panel of immunohistochemical marker(s) can distinguish between GCC and NGCC with high precision. In difficult cases, tissue genotyping should be employed to confirm the diagnosis [15].

The only definitive way to distinguish between GCC and NGCC is the demonstration of paternal genetic material in the tumor tissue by genetic profiling. The absence of paternal genetic components can diagnose NGCC by exclusion [15].

The primary standard management for NGCC is different from GCC as surgical excision is usually preferred. Often, these patients do not require comprehensive surgical staging, and lymphadenectomy and omentectomy can be omitted, if the omentum and lymph nodes grossly appear tumor-free. With regard to patients for whom fertility-sparing surgery is indicated with unilateral disease, contralateral ovary sampling is not recommended. This might lead to premature ovarian failure, particularly if postoperative chemotherapy is needed [16].

The management chemotherapy options and prognosis for the two types of choriocarcinoma are also quite different. The preferred chemotherapy regimen for GCC is methotrexate-based, either as monotherapy or in combination with etoposide, methotrexate, actinomycin D, cyclophosphamide, and vincristine (EMA/CO). For NGCC, however, the preferred chemotherapy regimen is three to six cycles of bleomycin, etoposide, and cisplatin [17,18].

This patient was treated with total abdominal hysterectomy with bilateral salpingo-oophorectomy followed by the BEP protocol. Similar case reports showed effective results by using cytoreductive surgery followed by chemotherapy with no evidence of disease after close follow-up [19].

As all choriocarcinomas secrete HCG, monitoring patients by measuring HCG's serum level during and after treatment completion along with CT of the chest, abdomen, and pelvis is crucial to check responsiveness and exclude recurrent disease. Most studies followed up patients for at least two years after the normalization of HCG [6,20].

## Conclusions

Gynecologists need to maintain a high index of suspicion for any malignant ovarian tumor, especially when a patient is presenting with persistent pelvic pain, as it may present in patients with no cachectic symptoms. The initial testing that should be done is ultrasound; however, MRI remains the most accurate to identify specific features of the malignancy. Tumor markers which include CA125, lactate dehydrogenase (LDH), alpha-fetoprotein (AFP), and HCG are essential for malignancy confirmation in perimenopausal women, allowing them to be referred to a tertiary care center for early proper intervention and to decrease patient's morbidity. 

Although GCC are more common than NGCC, care must be taken to have them in the differential diagnosis in patients with vascular mass and high HCG. We reported the first case of NGCC from Saudi Arabia that was treated with surgery after diagnosis followed by chemotherapy (BEP regimen).
